# Efficacy of liraglutide in treating type 2 diabetes mellitus complicated with non-alcoholic fatty liver disease

**DOI:** 10.1042/BSR20181304

**Published:** 2018-12-21

**Authors:** Feng Tian, Zhigang Zheng, Damin Zhang, Si He, Jie Shen

**Affiliations:** 1 Department of Endocrinology and Metabolism, Shenzhen Guangming New District People’s Hospital, Shenzhen 518000, China; 2 Department of Internal Medicine, Shenzhen Yantian District People’s Hospital, Shenzhen 518000, China; 3 Department of Endocrinology and Metabolism, The Third Affiliated Hospital of Southern Medical University, Guangzhou 510500, China

**Keywords:** Liraglutide, Metformin, Non-alcoholic fatty liver disease, Type 2 diabetes mellitus

## Abstract

Type 2 diabetes mellitus (T2DM) complicated with non-alcoholic fatty liver disease (NAFLD) is difficult to treat. The present study explored the efficacy of (liraglutide) Lira in treating T2DM complicated with NAFLD. A total of 127 patients suffering from T2DM complicated with NAFLD were enrolled in the present study, and randomly assigned to a Lira group (liraglutide injection: 0.6–1.2 mg/day, 12 weeks, *n*=52) or a Metformin (Met) group (oral metformin: 1000–1500 mg/day, 12 weeks, *n*=75). During the treatment phase, the values for fasting plasma glucose (FPG), 2 h plasma glucose (2hPG), glycated hemoglobin (HbA1c), aspartate aminotransferase (AST)/alanine aminotransferase (ALT), and adiponectin (APN) decreased in both the Lira and Met groups, and the levels of Δ2hPG, ΔAST/ALT, and ΔAPN in the Lira group were significantly lower than those in the Met group. The values for total cholesterol (TC), triglycerides (TG), low-and high-density lipoproteins (LDL and HDL), ALT, AST, weight, body mass index (BMI), waist to hip ratio (WHR), and C-reactive protein were markedly increased in both groups, and levels of ΔAST, ΔALT, Δweight, ΔBMI, ΔWHR, and ΔCRP (C-reactive protein) in the Lira group were significantly higher than those in the Met group. An analysis of treatment efficacy showed that liraglutide was better than metformin in its ability to significantly decrease the ALT levels in patients with combined T2DM and NAFLD. Furthermore, liraglutide was more effective than metformin at ameliorating the severity of T2DM complicated with NAFLD, and produced its effects by alleviating liver inflammation and improving liver function.

## Introduction

Type 2 diabetes mellitus (T2DM) is a common health problem worldwide, and its incidence has increased in recent decades [1]. Approximately 415 million individuals suffered from T2DM in 2015, and this number is predicted to reach 642 million by 2040 [2]. Non-alcoholic fatty liver disease (NAFLD) is a common metabolic disease associated with T2DM, insulin resistance, and other metabolic diseases [3,4]. NAFLD occurs in 70–90% of patents with T2DM, making it an important worldwide public health problem [5,6]. Although the treatment of T2DM has markedly changed in recent years, the best treatment for T2DM complicated with NAFLD remains unknown [7]. Therefore, it is important to explore new methods for treating NAFLD in patients with T2DM.

Liraglutide (Lira) is an analog of the incretin hormone glucagon-like peptide (GLP-1), and widely used in the treatment of T2DM [8,9]. Studies have revealed that liraglutide ameliorates T2DM by stimulating the secretion of insulin to promote glucose metabolism without producing significant hypoglycemia [10]. Moreover, liraglutide is also used to lower weight and blood pressure and reduce the risk for cardiovascular events in T2DM patients [11–13]. A previous study showed that NAFLD and T2DM patients share some pathophysiological characteristics [14], such as insulin resistance, abnormal insulin secretion, epigenetic alterations, glucose and lipid metabolism disorders, and factors related to lifestyle [14], suggesting that liraglutide can also be used for treating NAFLD. Experimental evidence suggests that a GLP-1 agonist might improve liver metabolism and reduce food intake [15,16]. A long-term study showed that liraglutide was safe and efficacious for treating non-alcoholic steatohepatitis, when compared with treatment with a placebo [17]. Moreover, a study by Kahal et al. [18] indicated that liraglutide might be useful for treating NAFLD [18]. However, studies on the use of liraglutide in treatment of combined T2DM and NAFLD have been rarely reported.

To explore the efficacy of liraglutide, patients with combined T2DM and NAFLD were enrolled in the present study and treated with liraglutide. Metformin (Met), a common pharmacological agent used for treating T2DM complicated with NAFLD [19], was used as a control drug. Various indicators of treatment efficacy were measured and compared between the liraglutide and metformin groups. We hope that our study results help clarify how to best use liraglutide in treating patients with combined NAFLD and T2DM.

## Materials and methods

### Patient selection

From January 2012 to December 2013, the present study enrolled 129 T2DM patients with NAFLD at Yangang Hospital in China. The protocol for the present study was approved by the Institutional Ethics Committee of Guangming District Central Hospital (Shenzhen, China). Patients were selected if they met the following criteria: (i) T2DM was diagnosed in accordance with criteria provided by the American Diabetes Association; i.e. fasting glucose ranged from 7.0–10.0 mmol/l, and glycated hemoglobin (HbA1c) was <9.0%; (ii) NAFLD was diagnosed in accordance with the Standard for NAFLD diagnosis recommended by the Fatty Liver and Alcoholic Liver Disease Group of the Chinese Medical Association in 2010, and confirmed by B-mode ultrasonic scanning; (iii) patient age in the range of 21–70 years; (iv) patient agreed to participate in the study. Patients were excluded if they met any of the following criteria: (i) a long-term drinking history or alcohol consumption ≥140 g/per week for males or ≥140 g/per week for females; (ii) NAFLD-associated diseases, including virus hepatitis, drug-induced liver disease, Wilson’s disease, and total parenteral nutrition; (iii) NAFLD and T2DM complicated with severe heart failure, infection, fever, or some other acute diseases; (iv) hepatic function indices that showed a >2.5-fold change from the maximum normal value; (v) serum creatinine >150 mmol/l or a creatinine clearance rate <60 ml/min; (vi) glutamic acid decarboxylase antibody (GAD-Ab) or insulin antibody positive; (vii) taking any of the following drugs for >2 weeks: diabetes-related drugs, insulin, or a GLP-analog, or receptor agonist; (viii) NAFLD and TG2DM complicated with an endocrine system disease, autoimmune disease, or desmosis; (ix) drug allergy, drug abuse, or receiving glucocorticoid or immunosuppressive therapy; (x) history of acute or chronic pancreatitis or pancreatectomy; (xi) pregnant or nursing females, or persons with other reasons for not participating in the study.

### Grouping and treatment

All patients received a diabetic diet and exercise education after enrolling in the study. The patients were then assigned to two groups (Met group, *n*=75 and Lira group, *n*=52) based on their treatment regimen. Patients in the Met group received Met at a dose of 100–1500 mg/day, while patients in the Lira group received Lira at a dose of 0.6–1.2 mg/day. The patients in both groups were treated for 12 weeks; after which, their clinical symptoms before and after treatment were compared.

### Anthropometric evaluation

Before and after treatment, primary and anthropometric estimations were made for all the enrolled patients. The parameters recorded included age, gender, weight, height, systolic blood pressure (SBP), diastolic blood pressure (DBP), body mass index (BMI), waist and hip circumferences, and waist to hip ratio (WHR). BMI was calculated as weight/height squared (kg/m^2^) and WHR was calculated as the waist circumference/hip circumference.

### Laboratory evaluation

Various laboratory indices were also measured before and after treatment for all the patients. Briefly, fasting venous blood samples were collected on the second morning to determine their levels of glucose (fasting plasma glucose (FPG) and 2hPG), HbA1c, triglycerides (TG), low-density lipoprotein (LDL), high-density lipoprotein (HDL), total cholesterol (TC), C-reactive protein (CRP), aspartate aminotransferase (AST), alanine aminotransferase (ALT), and insulin (Fins). All measurements were performed in a blinded manner by independent medical technicians who used a conventional automated analyzer in the biochemistry laboratory of our hospital. Additionally, adiponectin (APN) levels were determined using an ELISA kit (Cat.: DRP300; R&D, Wiesbaden-Nordenstadt, Germany) according to the manufacturer’s protocol. The homeostasis model assessment-insulin resistance index (HOMA-IR) was calculated as FPG × Fins/22.5. Differences in the index before and after treatment were calculated as Δindex = after_index_ − before_index_.

### Statistical analyses

All statistical analyses were performed using SPSS for Windows, Version 13.0 (SPSS Inc., Chicago, IL, U.S.A.). Results for continuous variables are presented as the mean ± S.D. and results for categorical variables are presented as a number and percentage (%). Comparisons of continuous variables between groups were performed using Student’s *t* test, and values for non-parameter continuous variables were first transformed into a natural logarithm and then compared using Student’s *t* test. Categorical variables were compared using the χ^2^ test. Differences within groups during intervention (Δ) were calculated as the final value minus the baseline value of each index, and comparisons between groups were performed using Student’s *t* test for paired samples. For all comparisons, a *P*-value <0.05 was considered to be statistically significant.

## Results

### Baseline characteristics of the enrolled patients

A total of 129 patients were enrolled in the present study, including 53 patients in the Lira group and 76 in the Met group. During the intervention, two patients (one in each group) terminated their treatment due to severe gastrointestinal reactions. Thus, a total of 127 patients (52 in the Lira group and 75 in the Met group) completed treatment. There were no significant differences in age, gender, SBP/DBP, weight, BMI, or WHR between the two groups (*P*>0.05). Moreover, the two groups showed no significant differences in values for TC, TG, LDL, HDL, ALT, AST, FPG, 2hPG, HbA1c, HOMA-IR, APN, and CRP (*P*>0.05, Table 1).

**Table 1:** Baseline characteristics of patients in the Lira and Met groups

Term	Lira group (*n*=52)	Met group (*n*=75)	*t*	*P*
Age (year)	58.5 ± 7.6	56.4 ± 8.4	−1.68	0.10
Gender ratio (male/female)	31/21	43/32	0.018	0.89
SBP (mmHg)	140 ± 20	138 ± 16	−0.072	0.943
DBP (mmHg)	91 ± 13.6	84 ± 12.6	−0.712	0.481
Weight (kg)	66.91 ± 10.03	66.27 ± 10.00	0.062	0.951
BMI (kg/m^2^)	28.18 ± 1.86	27.61 ± 1.77	1.58	0.12
TC (mmol/l)	5.03 ± 0.97	5.15 ± 0.92	−0.62	0.54
TG (mmol/l)	2.47 ± 1.49	2.51 ± 1.73	−1.01	0.32
LDL (mmol/l)	2.56 ± 0.83	2.62 ± 0.82	−1.57	0.12
HDL (mmol/l)	1.05 ± 0.24	0.99 ± 0.20	1.34	0.18
ALT (U/l)	65.74 ± 18.13	65.81 ± 17.58	−0.02	0.99
AST (U/l)	35.96 ± 12.27	34.28 ± 13.69	0.62	0.54
AST/ALT	0.60 ± 0.06	0.60 ± 0.05	0.05	0.96
FPG (mmol/l)	8.45 ± 0.64	8.41 ± 0.69	0.34	0.73
2hPG (mmol/l)	15.09 ± 5.42	14.64 ± 5.12	0.81	0.42
HbA1C (%)	8.14 ± 0.51	8.09 ± 0.59	−0.86	0.59
HOMA-IR	3.05 ± 0.55	2.94 ± 0.59	0.95	0.34
APN (mg/l)	8.43 ± 1.01	8.13 ± 1.23	1.27	0.21
CRP	3.14 ± 0.58	3.16 ± 0.68	−0.18	0.86
CK18 (IU/l)	284.03 ± 45.67	293.15 ± 38.55	1.26	0.23

Abbreviation: CK18, cytokeratin 18.

### Changes in indices after treatment for 12 weeks

All clinical indices were examined after 12 weeks of treatment, at which time, the Lira and Met groups showed no significant differences in their values for FPG, TC, TG, LDL, HDL, and HOMA-IR (all *P*-values >0.05, Table 2). However, the values for 2hPG, ALT, weight, BMI, WHR, and hs-CRP in the Lira group were significantly lower than those in the Met group (*P*<0.05), and the values for AST, AST/ALT, and APN in the Lira group were significantly higher than those in the Met group (*P*<0.05) (Table 2). During the treatment, no hypoglycemia events were identified in both groups. However, there were 13 patients who suffered mild and median intestinal side reaction, including 9 cases in the Lira group and 4 cases in the Met group (Table 2).

**Table 2:** Variations of indices after treatment for 12 weeks

Term	Lira group (*n*=52)	Met group (*n*=75)	*t*	*P*
Weight (kg)	76.09 ± 9.85	77.22 ± 10.15^1^	−2.764	0.02
BMI (kg/m^2^)	25.87 ± 1.49	26.91 ± 1.73^1^	−2.28	0.02
WHR	0.95 ± 0.05	0.96 ± 0.04^2^	−2.59	0.009
TC (mmol/l)	4.88 ± 1.51	4.82 ± 0.92	−0.09	0.954
TG (mmol/l)	1.84 ± 0.60	1.78 ± 0.51	0.95	0.325
LDL (mmol/l)	2.46 ± 0.53	2.45 ± 0.55	−0.029	0.687
HDL (mmol/l)	1.05 ± 0.24	1.03 ± 0.20	1.034	0.081
ALT (U/l)	39.82 ± 14.05	51.48 ± 18.89^2^	−3.36	0.000
AST (U/l)	25.61 ± 7.87	31.54 ± 10.75^1^	2.62	0.012
AST/ALT	0.81 ± 0.17	0.69 ± 0.15^2^	4.34	0.00
FPG (mmol/l)	7.11 ± 0.68	7.32 ± 0.76	−1.52	0.131
2hPG (mmol/l)	10.31 ± 3.17	12.05 ± 4.03	−2.43	0.024*
HbA1C (%)	7.23 ± 0.68	7.20 ± 0.37	0.32	0.749
HOMA-IR	2.47 ± 0.44	2.48 ± 0.51	−0.10	0.923
APN (mg/l)	10.44 ± 3.29	8.43 ± 2.31^2^	3.45	0.000
CRP (mg/l)	2.18 ± 0.34	2.69 ± 0.53^1^	−3.21	0.023
CK18 (IU/l)	228.36 ± 41.09	217.05 ± 37.84	1.60	0.112
Side effect (number)	9	4	-	-

Compared with the Lira group. Abbreviation: CK18, cytokeratin 18. ^1^*P*<0.05. ^2^*P*<0.01.

### Changes in the indices during intervention

The changes that occurred in indices during intervention were also calculated and compared between the Lira and Met groups. These comparisons showed that the values for FPG, 2hPG, and HbA1C were decreased after treatment for 12 weeks; however, the value for Δ2hPG in the Lira group was significantly lower than those in the Met group, while there was significant difference in the ΔHbA1C and ΔFPG values of the two groups (Table 3), indicating that liraglutide was better than metformin at alleviating the symptoms of T2DM. Moreover, values for AST/ALT and CRP had also decreased in both the Lira and Met groups, and the values for ΔAST/ALT and ΔCRP in the Lira group were significantly lower than those in the Met group (Table 3). On the other hand, the values for TC, TG, LDL, HDL, ALT, AST, weight, BMI, WHR, HOMA-IR, and CRP had increased after treatment for 12 weeks in both the Lira and Met groups. Specifically, the values for ΔALT, ΔAST, Δweight, ΔBMI, ΔWHR, and ΔCRP in the Lira group were significanly higher than those in the Met group (*P*<0.05, Table 3).

**Table 3:** Variations of indices during intervention

Term	Lira group (*n*=52)	Met group (*n*=75)	*t*	*P*
ΔWeight (kg)	4.16 ± 5.32	1.98 ± 3.28^2^	2.01	0.000
ΔBMI (kg/m^2^)	−1.31 ± 0.98	−0.69 ± 0.94^2^	3.22	0.000
ΔWHR	0.04 ± 0.02	0.02 ± 0.01^2^	3.08	0.000
ΔTC (mmol/l)	−0.29 ± 0.71	−0.26 ± 0.80	0.17	0.939
ΔTG (mmol/l)	−0.31 ± 1.44	−0.33 ± 1.06	−0.11	0.904
ΔLDL (mmol/l)	−0.19 ± 0.63	−0.16 ± 0.71	0.20	0.646
ΔHDL (mmol/l)	0.02 ± 0.50	0.03 ± 0.61	0.10	0.827
ΔALT (U/l)	−27.32 ± 15.96	−15.85 ± 11.38^2^	5.73	0.0002
ΔAST (U/l)	−7.89 ± 7.87	−6.98 ± 5.11^1^	2.03	0.048
ΔAST/ALT	0.23 ± 0.08	0.11 ± 0.09^1^	−1.02	0.043
ΔFPG (mmol/l)	−1.09 ± 0.83	−1.12 ± 0.80	0.08	0.581
Δ2hPG (mmol/l)	−4.16 ± 3.01	−2.63 ± 2.28	2.03	0.022^1^
ΔHbA1C (%)	−0.91 ± 0.65	−0.89 ± 0.57	1.30	0.203
ΔHOMA-IR	−0.57 ± 0.36	−0.56 ± 0.49	0.89	0.367
ΔAPN (mg/l)	2.22 ± 1.86	1.30 ± 0.76^2^	−7.37	0.001
ΔCRP (mg/l)	−0.89 ± 0.59	−0.61±0.53^1^	2.59	0.018

Compared with the Lira group. ^1^*P*<0.05 ^2^*P*<0.01.

### Treatment efficacy after treatment

The efficacy of treatment for NAFLD was estimated using B-mode ultrasound and a normal ALT level of <40 units/l. A normal reduced glucose level was defined as an HbA1C value <7%. As a result, the percentages of NAFLD patients in the Lira and Met groups were markedly lower when estimated by B-mode ultrasound (78.8 compared with 89.3%), but did not significantly differ in the two groups (*P*=0.13, Table 4). Moreover, total 19 and 33 patients in the Lira and Met groups, respectively, reached a normal HbA1C level of <7%, and the percentage of patients with normal HbA1C levels did not significantly differ in the two groups (36.5 compared with 44%, *P*=0.62, Table 4). In addition, 29 patients in the Lira group and 20 patients in the Met group reached a normal ALT level of <40 units/l, and this target rate in the Lira group was significantly higher than that in the Met group (55.8 compared with 26.7%, *P*=0.02, Table 4). No patient in either group developed glucopenia during treatment; however, nine patients in the Lira group and four patients in the Met group experienced slight to moderate gastrointestinal disturbances.

**Table 4:** Treatment efficacy assessed by B-mode ultrasound, HbA1C, and ALT (*n*(%))

Group	Non-NAFLD	NAFLD	HbA1C <7	HbA1C >7	ALT <40	ALT >40
Lira group	11	41	19	33	29	23
(*n*=52)	21.2%	78.8%	36.5%	63.5%	55.8%	44.2%
Met group	8	67	33	42	20	55
(*n*=75)	10.7%	89.3%	44%	56%	26.7%	73.3%
χ^2^	2.35		0.24		9.86	
*P*	0.13		0.62		0.02	

### Liraglutide therapeutic effect associated clinical factors

To explore liraglutide therapeutic effect associated clinical factors, liraglutide treated patients were divided into the ALT <40 (*n*=29) and ALT ≥40 (*n*=23) groups according to improved condition after treatment. Then, comparisons were performed between these two groups about the associated clinical factors. The results presented no significant differences between these two groups in the age, gender, weight, TG, HDL, and HbA1C of patients (all *P*>0.05), while BMI, WHR, TC, LDL, FPG, 2hPG, HOMA-IR, APN, and CRP were significantly correlated with the improved condition of T2DM patients complicated with NAFLD (all *P*<0.05, Table 5). Therefore, it is important to take more consideration on levels of BMI, WHR, TC, LDL, FPG, 2hPG, HOMA-IR, APN, and CRP of T2DM patients complicated with NAFLD treated by liraglutide.

**Table 5:** Variations of indices in ALT <40 and ALT >40 group after Lira treatment for 12 weeks

Term	ALT <40 (*n*=29)	ALT >40 (*n*=23)	*t*	P
Age (year)	57.3 ± 5.8	60.1 ± 4.9	−1.85	0.07
Gender ratio (male/female)	16/14	15/8	-	0.53^3^
Weight (kg)	75.33 ± 5.66	78.92 ± 4.56	−1.78	0.08
BMI (kg/m^2^)	24.93 ± 1.02	27.05 ± 0.86^2^	−7.97	0.000
WHR	0.93 ± 0.04	0.98 ± 0.07^1^	−2.19	0.033
TC (mmol/l)	4.72 ± 0.63	5.08 ± 0.46^1^	−2.30	0.026
TG (mmol/l)	1.78 ± 0.21	1.93 ± 0.37	−1.84	0.071
LDL (mmol/l)	2.39 ± 0.29	2.55 ± 0.27^1^	−2.04	0.047
HDL (mmol/l)	1.02 ± 0.24	1.09 ± 0.21	−1.10	0.275
FPG (mmol/l)	6.75 ± 0.33	7.56 ± 0.34^2^	−8.67	0.000
2hPG (mmol/l)	9.75 ± 1.79	11.03 ± 2.52^1^	−2.41	0.037
HbA1C (%)	7.14 ± 0.45	7.35 ± 0.57	−1.49	0.144
HOMA-IR	2.29 ± 0.35	2.69 ± 0.38^2^	−3.94	0.000
APN (mg/l)	9.48 ± 2.15	10.87 ± 2.04^1^	−2.37	0.021
CRP (mg/l)	2.07 ± 0.19	2.32 ± 0.22^2^	−4.39	0.000

Compared with the ALT <40 group. ^1^*P*<0.05 . ^2^*P*<0.01 (unpaired *t* test). ^3^Chi-square test.

## Discussion

As society has developed, the quality of life of individuals has markedly improved, however the amount of physical exercise performed by individuals has significantly decreased. Along with these changes, the occurrences of NAFLD and T2DM have significantly increased, as well as the prevalence of T2DM complicated with NAFLD, which is considered to be an important risk factor for cardiovascular diseases [20,21]. Previous studies have documented that incretin-based treatment improves fatty liver and various indicators of liver dysfunction [22–24]. In the current study, we showed that 12 weeks of liraglutide treatment could significantly improve several metabolic parameters and alleviate NAFLD symptoms in T2DM patients.

Metformin is a first-line pharmacological therapy for T2DM and works by reducing glucose output and promoting glucose uptake in the peripheral tissues of diabetic patients [25]. Moreover, metformin also regulates hepatic lipid metabolism by activating AMP-activated protein kinase [26]. Several studies have suggested that metformin might be beneficial in treating NAFLD [27–29]. In the present study, we found that metformin could moderately improve the clinical parameters of T2DM patients with NAFLD. However, a study by Garinis et al. [30] showed that 6 months of treatment with metformin did not improve clinical parameters in cases of NAFLD [30]. This difference in results might be attributable to certain demographic characteristics of the patients in the two studies and (or) differences in sample size. A meta-analysis of studies that used metformin in treating NAFLD might be required in the future. Liraglutide is a strong GLP-1 receptor agonist and widely used in treating T2DM [31]. Studies have shown that GLP-1 agonists bind to the receptor of the endogenous, intestinally secreted hormone GLP-1 to enhance insulin secretion. Such binding reduces gastric emptying and food intake, inhibits the production of postprandial glucagon, and thereby suppresses the progression of fatty liver disease in patients with T2DM [32]. Based on the evidence described above, both liraglutide and metformin are promising drug candidates for treating T2DM complicated with NAFLD.

In the current study, the levels of ΔFPG, Δ2hPG, ΔHbA1c, and ΔHOMA-IR were reduced in both the Lira and Met groups after 12 weeks of treatment; however, the Δ2hPG values in the Lira group were significantly lower than those in the Met group (*P*<0.05), indicating that treatment with liraglutide was more effective than treatment with metformin. When compared with metformin, liraglutide not only promoted insulin secretion, but also inhibited glucagon release and delayed emptying of the stomach [33]. Thus, we deduced that this effect might explain the difference between the Lira and Met groups. However, there were no significant differences in the values for ΔHOMA-IR and ΔHbA1C in the Met and Lira groups. We suspect that long-term treatment of a larger number of subjects might be required to further confirm these findings.

Weight loss is an important indicator for the treatment of T2DM [34]. In the current study, the values for weight, BMI, and WHR were also decreased in both the Lira and Met groups, and levels of Δweight, ΔBMI, and ΔWHR in the Lira group were significantly lower than those in the Met group. A previous short-term analysis showed that liraglutide was superior to metformin for inducing weight loss in obese patients with polycystic ovary syndrome (PCOS) [35]. Metformin is frequently used to treat obesity due to its clinical benefits, which include body weight reduction and fat distribution arrangement in patients with PCOS [36]. Nauck et al. [37] also reported that liraglutide was superior to metformin for producing weight loss in T2DM patients [37]. Despite these findings, other studies have demonstrated that metformin cannot significantly reduce body weight [38,39], which is consistent with the findings in our current study. Taken together, the existing evidence indicates that liraglutide might be better than metformin at producing weight loss in patients with combined T2DM and NAFLD. However, there were no differences in the levels of TC, TG, LDL, and HDL, as well as the NAFLD percentages as diagnosed by B-Mode ultrasound between the Lira and Met groups; hence, further studies are needed. APN is an insulin-sensitive adipocytokine that plays critical roles in adipose tissue inflammation [40]. APN can inhibit NAFLD by reducing fat content and promoting fatty acid oxidation; it also inhibits liver inflammation by attenuating TNF-α levels [41]. A low serum APN indicates an advanced fibrosis condition in patients with NAFLD [42]. Chinese herb extract improves steatosis in NAFLD rats via increasing the expression of APN. A recent study had further explored that APN could protect obesity or diabetes-induced NAFLD via activating AdipoRs/PPARα signaling pathway [43]. In the current study, liraglutide significantly elevated the levels of APN when compared with the effect produced by metformin, indicating that liraglutide produced a greater beneficial effect on the liver in patients with combined T2DM and NAFLD. Moreover, CRP is another biomarker for systematic inflammation in humans, and can be up-regulated by insulin via the IRS/PI3K signaling pathway in patients with NAFLD [44]. High level of CRP predicts the development of NAFLD [45]. In addition, high level of CRP was also used for the prediction of metabolic syndrome, T2DM, and coronary heart disease [46,47]. Despite the controversial opinions, there are still several studies identified the association between high levels of CRP and NAFLD [48–50]. In this study, liraglutide was more effective than metformin at decreasing the levels of CRP. Furthermore, the levels of AST and ALT were also significantly lower in the Lira group when compared with those in the Met group, which was consistent with the changes observed in APN and CRP levels. This combined evidence indicates that liraglutide can improve liver function by reducing inflammation and metabolic syndromes in NAFLD.

In conclusion, both liraglutide and metformin could ameliorate the abnormal glucose and lipid metabolism, as well as the inflammation found in patients with combined T2DM and NAFLD. Moreover, liraglutide was more effective than metformin at promoting weight control, reducing blood glucose levels, and alleviating inflammation in patients with combined T2DM and NAFLD. Therefore, liraglutide displayed a better therapeutic profile for treating T2DM complicated with NAFLD. However, due to the small sample size and short treatment duration in the present study, the comparative effects of these two drugs on HOMA-IR and fat distribution remain unclear, and further investigations are needed.

## Highlights

1. A total of 127 patients with T2DM complicated with NAFLD were treated with liraglutide or metformin for 12 weeks.
2. Liraglutide decreased the levels of FPG, 2hPG, HbA1c, APN, and AST/ALT.
3. Liraglutide was better than metformin at ameliorating the severity of T2DM complicated with NAFLD.

## Abbreviations

ALT: alanine aminotransferase
APN: adiponectin
AST: aspartate aminotransferase
BMI: body mass index
CRP: C-reactive protein
DBP: diastolic blood pressure
FPG: fasting plasma glucose
GLP-1: glucagon-like peptide
HbA1c: glycated hemoglobin
HDL: high-density lipoprotein
HOMA-IR: homeostasis model assessment-insulin resistance index
hs-CRP: high sensitvity C-reactive protein
IRS: insulin receptor substrate
LDL: low-density lipoprotein
NAFLD: non-alcoholic fatty liver disease
PCOS: polycystic ovary syndrome
PI3K: phosphatidylinositol 3-kinase
SBP: systolic blood pressure
TC: total cholesterol
TG: triglyceride
TNF-a: tumor necrosis factor-a
T2DM: Type 2 diabetes mellitus
WHR: waist to hip ratio
2hPG: 2 h plasma glucose
